# Dynamic Monitoring of sTREM-1 and Other Biomarkers in Acute Cholangitis

**DOI:** 10.1155/2020/8203813

**Published:** 2020-05-15

**Authors:** Jiahui Jiang, Xiaolei Wang, Tongtong Cheng, Mingyue Han, Xinxin Wu, Haitong Wan, DaojunYu

**Affiliations:** ^1^Affiliated Hangzhou First People's Hospital, Zhejiang Chinese Medical University, Hangzhou, China; ^2^Department of Clinical Laboratory, Affiliated Hangzhou First People's Hospital, Zhejiang University School of Medicine, Hangzhou, China; ^3^Zhejiang Chinese Medical University, Hangzhou, China

## Abstract

**Background:**

Sepsis is a common complication of acute cholangitis (AC), which is associated with a high mortality rate. Our study is aimed at exploring the significance of white blood cell (WBC), C-reactive protein (CRP), procalcitonin (PCT), soluble triggering receptor expressed on myeloid cells 1 (sTREM-1), and temperature (T) alone or combined together in early identification and curative effect monitoring of AC with or without sepsis.

**Methods:**

65 consecutive cases with AC and 76 control cases were enrolled. They were divided into three groups: Group A (AC with sepsis), Group B (AC without sepsis), and Group C (inpatients without AC or other infections). The levels of WBC, CRP, PCT, sTREM-1, and temperature were measured dynamically. The study was carried out and reported according to STARD 2015 reporting guidelines.

**Results:**

CRP had the highest AUC to identify AC from individuals without AC or other infections (AUC 1.000, sensitivity 100.0%, specificity 100.0%, positive predictive value 100.0%, and negative predictive value 100.0%). Among various single indexes, PCT performed best (AUC 0.785, sensitivity 75.8%, specificity 72.2%, positive predictive value 68.7%, and negative predictive value 78.8%) to distinguish sepsis with AC, while different combinations of indexes did not perform better. From day 1 to day 5 of hospitalization, the levels of sTREM-1 in Group A were the highest, followed by Groups B and C (*P* < 0.05); on day 8, sTREM-1 levels in Groups A and B declined back to normal. However, other index levels among three groups still had a significant difference on day 10. Both in Groups A and B, sTREM-1 levels declined fast between day 1 and day 2 (*P* < 0.05).

**Conclusions:**

CRP is the best biomarker to suggest infection here. PCT alone is sufficient enough to diagnose sepsis with AC. sTREM-1 is the best biomarker to monitor patients' response to antimicrobial therapy and biliary drainage.

## 1. Introduction

Biliary tract infection (BTI), including cholangitis and cholecystitis, occurs when there is biliary stenosis due to various benign causes (often bile duct stone) and malignant causes [1, 2]. Acute cholangitis (AC), especially acute suppurative cholangitis (ASC), easily progresses to a clinically severe condition, such as sepsis. Gram-negative bacteria are the most common organisms in acute cholangitis cases, accounting for about 76.6% [3] of the aforementioned cases. When the significant proliferation of bacteria in the bile duct increases the pressure in the bile duct, microorganisms or endotoxins are flushed into the systemic circulation and trigger a systemic inflammatory response [2, 4]. Sepsis caused by acute cholangitis is described as a compatible clinical syndrome and a blood culture isolate consistent with ascending cholangitis [5]. When AC with sepsis appears, unless appropriate biliary drainage and antibiotics are rapidly given, the systemic condition will suddenly deteriorate, which often results in mortality [4]. The 30-day mortality rate of patients with sepsis caused by AC has been reported to be ∼10% [6, 7]. In the Tokyo Guidelines, the selection of an empiric antimicrobial agent is dependent on severity of cholangitis [8]. Thus, assessing the severity of AC and diagnosing sepsis caused by AC as early as possible will guide practitioners to take appropriate and immediate treatment. Hemoculture is seen as an important method to diagnose sepsis, but it remains negative in more than half of cases [5, 9]. The bile culture may have a higher positivity rate [10], but the culture must be used after endoscopic retrograde cholangiopancreatography (ERCP), which is an invasive operation. There is an inevitable negative rate in the bile culture, and positive bile culture may not indicate the AC [9–11]. What is more, using either blood or bile culture costs too much time.

White blood cell (WBC), C-reactive protein (CRP), and temperature (T) are necessary parameters in systemic inflammation. TG18 (Tokyo Guideline 2018) also incorporates them in the diagnostic criteria of AC. In recent years, PCT, which can be detected earlier than CRP, has gradually become a specific blood marker of bacterial, parasite, and fungal infections [4]. Accumulating evidence indicates that the blood level of PCT can well predict the existence and severity of systemic infectious diseases [12, 13]. However, the levels of WBC, CRP, PCT, and T are elevated in many noninfectious diseases such as autoimmune and rheumatic disorders, major surgery, and severe burns [14]. There are still false positives and false negatives in the diagnosis of AC and sepsis by WBC, CRP, and PCT tests [7]. In recent studies, there are different opinions on the specificity and sensitivity of parameters mentioned above in patients with AC and patients with sepsis caused by AC: Beliaev et al. have found that lymphocyte count, neutrophil-to-lymphocyte ratio (NLR), and CRP have the highest discriminative powers to diagnose patients with AC, while WBC and albumin have the poorest [15]; Qin et al. have figured out that abnormal WBC and CRP are two independent risks for ASC, and a high level of CRP indicates the presence of severe sepsis [1]. Thus, more research should be undertaken to figure out how to diagnose AC and sepsis accurately and quickly.

Triggering receptor expressed on myeloid cells (TREM) 1 is an innate inflammatory transmembrane receptor mainly expressed on monocytes/macrophages and neutrophils [16]. After binding to the adaptor DNAX activation protein- (DAP-) 12, TREM-1 amplifies the signaling of Toll-like receptors (TLRs) TLR4 or TLR2, which can recognize components of a variety of microorganisms including bacteria and fungi [17]. The expression of TREM-1 will increase after infection [18], and some membranous TREM-1 is shed into the systemic circulation through the activation of metalloproteinases [19]. sTREM-1 (soluble TREM-1) is a potential marker to diagnose infectious diseases, as well as to evaluate the disease severity and clinical efficacy [20, 21]. However, there are limited studies on the influence of sTREM-1 levels on the early diagnosis and severity judgement of AC.

There is still a lack of accurate indexes to predict and diagnose sepsis in a timely manner. Hence, we conduct this prospective study to monitor some biomarkers (WBC, CRP, PCT, and sTREM-1) and body temperature (T) in AC patients with or without sepsis and control individuals. Thus, we would figure out the significance of these biomarkers and temperature alone or combined together in early diagnosis of AC and sepsis, as well as the prediction of treatment effect. We will estimate the value of sTREM-1 and other indicators alone or combined together in early identification and curative effect monitoring of AC with or without sepsis.

## 2. Methods

### 2.1. Patient Demography

In this prospective study, we enrolled consecutive patients of acute cholangitis (AC) in Affiliated Hangzhou First People's Hospital, Zhejiang University School of Medicine, from December 2018 to June 2019. Patients eligible for inclusion were adults (≥18years) with AC, based on the Tokyo Guideline 2018 diagnostic criteria for acute cholangitis [2]. We excluded patients if they received antibiotic therapy at presentation, if they were pregnant, if they were in the intensive care unit or haematology ward, or if follow-up was not feasible. Sepsis was defined as life-threatening organ dysfunction caused by a dysregulated host response to infection [22]. Patients with sepsis had either microbiologically (culture-proven) or clinically diagnosed sepsis. Microorganisms from positive blood and bile culture should be the same. The criteria proposed by the Society of Critical Care Medicine and the European Society of Intensive Care Medicine [22] were used to diagnose sepsis clinically. The baseline Sequential Organ Failure Assessment (SOFA) score could be assumed to be zero in patients not known to have preexisting organ dysfunction [22]. A SOFA score ≥ 2 points was considered to be sepsis. All cases were evaluated by 2 clinicians, and agreement about the diagnosis was achieved in all cases. All patients diagnosed AC were given empiric antibiotic therapy according to the Tokyo Guidelines 2018 [8] after admission. The blood for microbial culture was collected before antibiotic therapy. All patients were given bile drainage within 48 h, and the bile for microbial culture was collected after ERCP. Once the microbe sensitivity test was known, the appropriate antibiotic was selected. Study procedures were approved by the Hangzhou First People's Hospital ethics committee (approval number 2018-020-01) which waived the need for informed consent because biomarkers were measured on residual blood after completing a routine follow-up. As recommended, patients or their family were orally informed of a sample collection and of the purpose of this study.

All cases included were divided into two groups: Group A (AC with sepsis) and Group B (AC without sepsis). Patients with sepsis caused by factors unrelated to AC were excluded. We enrolled hypertensive and diabetic inpatients without AC or other infections as the control group (Group C).

Clinical, laboratory, and imaging data were recorded, including (1) age and gender, (2) past medical history and clinical presentation, (3) use of antibiotics, (4) bacterial culture of bile and blood, and (5) the body temperature on days 1, 2, 3, 5, 8, and 10 of hospitalization. On days 1, 2, 3, 5, 8, and 10 of hospitalization, venous blood anticoagulated with EDTA was drawn into two tubes. One tube was tested for WBC and CRP immediately; the other was centrifuged to take plasma and stored at -80°C until analysis.

### 2.2. Laboratory Tests

#### 2.2.1. WBC Assay

Two milliliters of peripheral blood samples anticoagulated with EDTA was obtained on certain days mentioned above. The whole blood cell count and white blood cell count (WBC) were measured by Mindray BC-6900 (Mindray, Shenzhen, China). Each sample was measured 3 times, and the average value was taken.

#### 2.2.2. CRP Assay

Two milliliters of peripheral blood samples anticoagulated with EDTA was obtained on certain days mentioned above. CRP levels were determined using a C-reactive protein kit (Mindray, Shenzhen, China) and detected by BC-5390 (Mindray, Shenzhen, China). Each sample was measured 3 times, and the average value was taken. The reference range for CRP measurements was 0.2–320 mg/L.

#### 2.2.3. PCT Assay

Each plasma frozen before was rewarmed to the room temperature. The levels of PCT were determined using a procalcitonin assay kit and measured by UPT-3A Analyzer (Hotgen Biotech, Beijing, China) according to the manufacturer's instructions. Each sample was measured 3 times, and the average value was taken. The reference range measured by the assay is 0.02-50 ng/mL.

#### 2.2.4. sTREM-1 Assay

Each plasma frozen before was rewarmed to room temperature. Plasma sTREM-1 was measured by commercial enzyme-linked immunosorbent assays (human triggering receptor expressed on myeloid cells 1 (TREM-1) ELISA kit; CUSABIO, Wuhan, China) according to the manufacturer's instructions. Each sample was measured 3 times, and the average value was taken. The reference range measured by the assay is 31.25-2000 pg/mL.

### 2.3. Statistical Analysis

Values are presented as the median (interquartile range). Comparisons between groups were made using a nonparametric test. Receiver operating characteristic (ROC) curves were established to evaluate the diagnostic value for discriminating among the three groups above. The area under curves (AUCs) with corresponding 95% confidence intervals (CI) and optimal cutoff values with sensitivity/specificity were calculated. The diagnostic values of different indexes and index combinations were compared by a two-sample *z* test. Probability values < 5% (*P* < 0.05) were considered statistically significant. Spearman rank correlation analysis was used to explore correlations between sTREM-1 and WBC in three groups. All statistics and graphs were prepared using SPSS for Windows (Statistical Package for the Social Sciences, IBM SPSS Statistics 20).

The study protocol was designed according to the recommendations of the Standards for Reporting of Diagnostic Accuracy Studies (STARD 2015) [23].

## 3. Results

### 3.1. Characteristics of the Study Subjects

**Figure 1:**
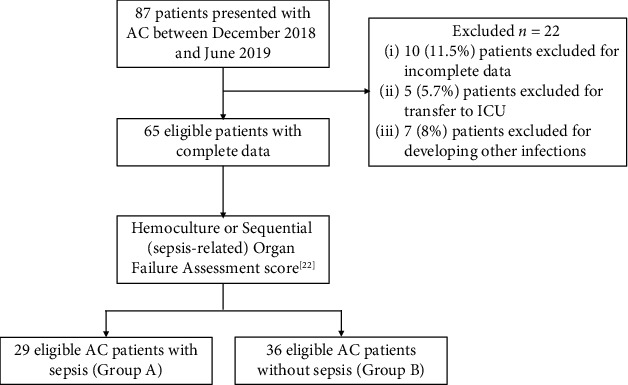
Flow chart showing the selection of patients for inclusion in the study.

A total of 87 patients with AC were evaluated for initial eligibility in this study. 10 (11.5%) patients were excluded for incomplete data, 5 (5.7%) patients were transferred to ICU due to worsening condition, and 7 (8.0%) patients were excluded for developing other infectious diseases (Figure 1). In this study, 65 eligible patients were analyzed and divided into two groups: Group A (AC with sepsis, *n* = 29) and Group B (AC without sepsis, *n* = 36). 76 inpatients without AC or other infectious diseases were enrolled as the control group (Group C). Detailed demographic data and comorbidities of the study population are summarized in Table 1.

### 3.2. Comparison of Blood Biomarker Levels and Temperature among the Three Groups

**Figure 2:**
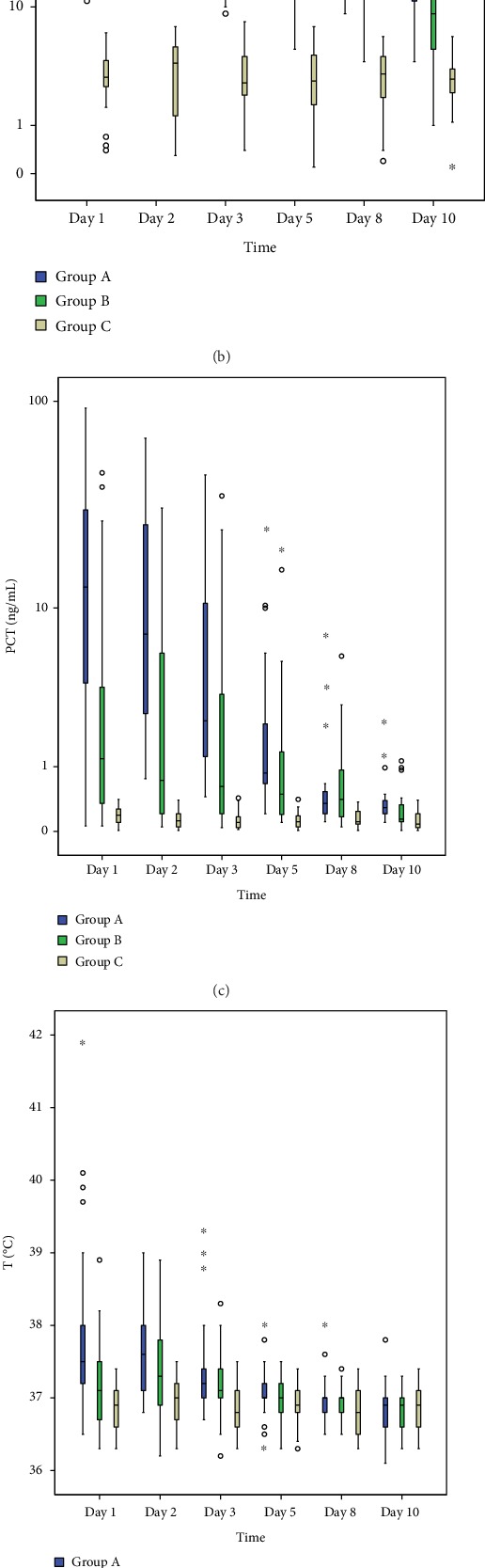
Dynamic monitoring of WBC, CRP, PCR, T, and sTREM-1 in three groups. WBC: white blood cell; CRP: C-reactive protein; PCT: procalcitonin; T: temperature; sTREM-1: soluble triggering receptor expressed on myeloid cells 1. Group A (blue), AC patients with sepsis; Group B (green), AC patients without sepsis; Group C (yellow), inpatients without AC or other infections. (a) The sTREM-1 levels among three groups had significant difference from day 1 to day 5. And both in Groups A and B, sTREM-1 levels declined fast between day 1 and day 2 (*P* < 0.05). (b) The WBC level in Group A was higher than that in Groups B and C from day 1 to day 5, and it was higher in Group B than in Group C. (c) CRP levels among three groups had significant difference for all days (*P* < 0.01), except CRP levels between Group A and Group B on day 1. (d) Group A had a significantly higher PCT level than Groups B and C in all days; and Group B's PCT level was higher than Group C's from day 1 to day 5. (e) On day 1 and day 3, Group A's T was higher than that of Groups B and C; on day 2, Group A and B's T was higher than Group C, while there was no difference between Groups A and B.

Laboratory values of WBC, CRP, PCT, T, and sTREM-1 in three groups of each day were listed in Table 2. From day 1 to day 5 of hospitalization, the levels of sTREM-1 in Group A were the highest, followed by Group B and Group C (*P* < 0.05) (Figure 2(a)). And on day 8, sTREM-1 levels in Groups A and B declined back to normal. What is more, both in Group A and Group B, there was a significant difference between the levels of sTREM-1 on day 1 and day 2 (*P* < 0.05). Compared with Group C, both Group A and Group B had higher levels of WBC from day 1 to day 5; and on day 8, levels of WBC in Group A were still significantly different from those of Group C (*P* < 0.05). However, the levels of WBC did not differ significantly between Group A and Group B for any days (Figure 2(b)). The levels of CRP among three groups had a significant difference in all days (*P* < 0.01), except that the CRP level in Group A had no difference with Group B on day 1 (Figure 2(c)). As for the levels of PCT, Group A had significantly higher levels than Group B and Group C in all days (*P* < 0.01); Group B's PCT levels were higher than those of Group C from day 1 to day 5 (*P* < 0.05) (Figure 2(d)). And on day 1 and day 3, the temperature of patients in Group A was higher than that of Group B and Group C (*P* < 0.05); and on day 2, the temperature of patients in Group A and Group B was higher than that of Group C, while there was no difference between Group A and Group B (Figure 2(e)). All the statistical information is shown in Figure 2.

### 3.3. Blood Biomarkers and Temperature for Diagnosing AC and Sepsis on the First Day

**Figure 3:**
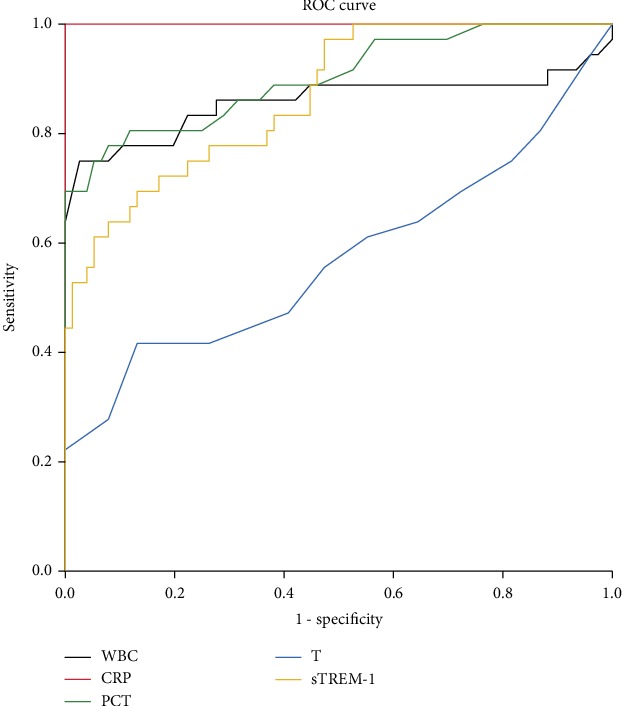
ROC curves of WBC, CRP, PCT, sTREM-1, PCT, and T between Group B and Group C. WBC (black): white blood cell; CRP (red): C-reactive protein; PCT (green): procalcitonin; sTREM-1 (yellow): soluble triggering receptor expressed on myeloid cells 1; T (blue): temperature. Group B: AC patients without sepsis; Group C: inpatients without AC or other infections.

**Figure 4:**
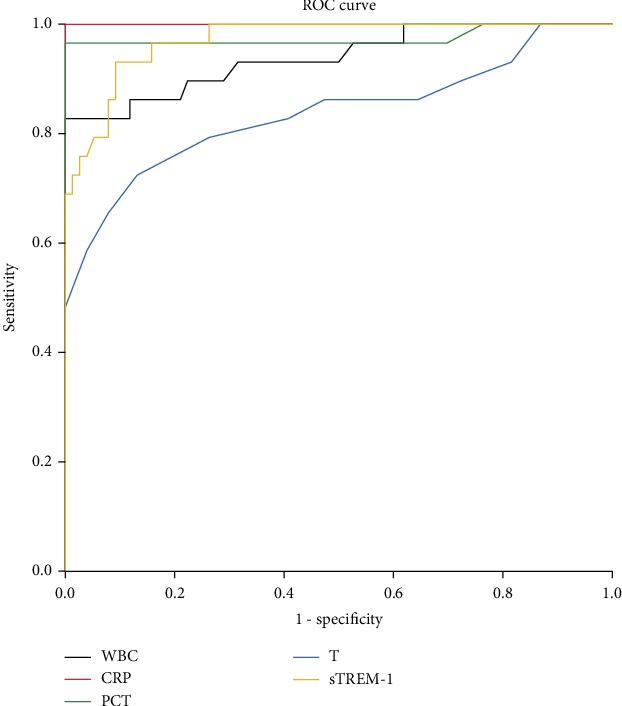
ROC curves of WBC, CRP, PCT, sTREM-1, PCT, and T between Group A and Group C. WBC (black): white blood cell; CRP (red): C-reactive protein; PCT (green): procalcitonin; sTREM-1 (yellow): soluble triggering receptor expressed on myeloid cells 1; T (blue): temperature. Group A: AC patients with sepsis; Group C: inpatients without AC or other infections.

To improve the diagnostic accuracy and reliability, it is better to use the values of biomarkers before treatment. Thus, we performed the ROC curve analysis to evaluate the potential value of these blood biomarker levels and temperature on the first day for diagnosing AC and sepsis. To diagnose AC without sepsis, CRP showed the highest AUC (*z* test, *P* < 0.05), followed by PCT, sTREM-1, and WBC (Figure 3). With 9.45 mg/L as the cutoff value, the sensitivity, specificity, positive predictive value (PPV), and negative predictive value (NPV) of CRP were all 100.0% (Table 3). CRP and PCT played a prominent role in diagnosing AC patients with sepsis from the healthy control (Figure 4 and Table 4). To diagnose AC with sepsis, the power of PCT was the highest (AUC 0.758, sensitivity 75.9%, specificity 72.2%, positive predictive value 68.7%, negative predictive value 78.8%, positive likelihood ratio 2.73, and negative likelihood ratio 0.33), followed by T and sTREM-1. Moreover, we analyzed different combinations of biomarkers to obtain the best performance. We found that different combinations of indexes did not enhance the diagnostic power (*z* test, *P* > 0.05), as shown in Table 5. In addition, WBC and CRP alone or combined together showed the lowest diagnostic power.

### 3.4. Correlations between WBC and sTREM-1 in Three Groups

Since sTREM-1 is mainly expressed by monocytes/macrophages and neutrophils, we further explored correlations between WBC and sTREM-1 in each group. In Group C, there was no correlation between WBC and sTREM-1 (*P* > 0.05). In Group A, the correlation coefficients (*r*) of WBC and sTREM-1 from day 1 to day 8 were between 0.4 and 0.8 (*P* < 0.01), and these correlation coefficients had no significant difference with each other (*P* > 0.05). In Group B, *r* of WBC and sTREM-1 from day 1 to day 3 were between 0.4 and 0.7 (*P* < 0.01), and these correlation coefficients had no significant difference with each other (*P* > 0.05). All the statistical information is shown in Table 6.

## 4. Discussion

Delays in diagnosis and treatment of acute cholangitis (AC) will exacerbate the infection and favour the development of sepsis, thus resulting in organ failure and threatening the lives of many. Nevertheless, overuse of antibiotics may contribute to the emergence of bacterial resistance. Therefore, accurate and timely identification of AC with or without sepsis is essential for the optimal management of patients, further limiting morbidity and improving the prognosis of patients.

WBC, CRP, and PCT have been widely used as biomarkers in nearly all studies of infection, but these biomarkers still have false positives and negatives in the diagnosis of infection and sepsis [7]. TREM-1 is a cell surface receptor expressed on myeloid cells. When exposed to bacteria and fungi, the expression of TREM-1 and the release of sTREM-1 will increase [18]. Recently, more and more studies have investigated the role of plasma sTREM-1 in differentiating infectious diseases [17, 24, 25].

It has been reported that acute suppurative cholangitis (ASC), the severe form of acute cholangitis, is associated with abnormal WBC and high CRP level [1]. The results of this study show that CRP is the best biomarker for biliary tract inflammation or infection (AUC 1.000, sensitivity 100.0%, specificity 100.0%, positive predictive value 100.0%, and negative predictive value 100.0%), followed by PCT, sTREM-1, and WBC, while T is the least accurate index (AUC 0.574, sensitivity 41.7%, specificity 86.8%, positive predictive value 60.0%, and negative predictive value 75.9%). Beliaev et al. have the same opinion that among common inflammatory markers for diagnosing AC, lymphocyte count, neutrophil-to-lymphocyte ratio (NLR), and CRP have the highest discriminative powers [15]. Usually, liver synthesis of CRP begins 4~6 hours and arrives at a peak at 36–50 h after the inflammation onset [14]. We consider that the great value of CRP in the differential diagnosis of AC with or without sepsis in this study might be associated to the fact that most patients do not seek medical advice in time, with a delay of 2-3 days when they have clinical symptoms like fever. Thus, the levels of CRP might be at the peak stage on the first day of hospitalization. Whereas, PCT is a sensitive and specific biomarker of systemic bacterial or fungal infection [13] and may not increase significantly in localized bacterial infections such as AC. Elderly patients or patients with severe infection occasionally have low to normal temperature and WBC [26]. Hence, these indexes show different values in diagnosis of AC in this study.

For the diagnosis of sepsis due to AC, our data indicates that PCT alone seems to be the best index (AUC 0.758, sensitivity 75.9%, specificity 72.2%, positive predictive value 68.7%, and negative predictive value 78.8%). sTREM-1 (AUC 0.737, sensitivity 58.6%, specificity 86.1%, positive predictive value 77.3%, and negative predictive value 72.1%) has almost similar diagnostic value of sepsis with PCT. In contrast, WBC and CRP alone or combined together do not show any advantage on the diagnosis of sepsis, whose AUCs are between 0.5 and 0.6. Loonen et al.'s study has demonstrated that PCT is significantly different in patients with positive blood cultures and negative blood cultures, while CRP levels remain similar in both groups [27]. Some recent research has also found that PCT concentrations at initial hospital presentation correlate well with AC severity and hemoculture positivity and might be used as an indicator for aggravation in patients with AC [4, 28, 29]. Kargaltseva et al. have found that when Gram-negative bacteremia occurs, the level of PCT increases, while the level of PCT stays normal in the case of coagulase-negative staphylococci bacteremia [30]. Since Gram-negative organisms, especially *Escherichia coli*, are the most common infecting organisms in AC, the PCT level is a helpful marker. Even so, since sepsis due to AC will also be caused by Gram-positive organisms, sTREM-1 should be taken into account, which will be upregulated by Toll-like receptor ligands such as lipoteichoic acid (LTA) of Gram-positive bacteria and lipopolysaccharide (LPS) of Gram-negative bacteria [31]. In Liao et al.'s study, the sensitivity of TREM-1 in peripheral blood mononuclear cells of sepsis patients with AC is higher than that of CRP and TNF-*α*, which is similar to our conclusion, but they have not tested PCT and WBC for comparison [32]. In spite of the little diagnostic value of sepsis on the first day, we find the continuation of the high CRP level correlated with the existence of sepsis: the CRP levels in Group A (AC with sepsis) are higher than those of other groups from day 2 to day 10 (*P* < 0.05). We assume that CRP synthesis in the liver might persist because of the longer treatment time of sepsis than local infection. In addition, the time course of CRP induction and clearance is slower than that of PCT [33]. So, the high CRP level maintains longer in sepsis patients in this study.

After giving the antibiotic therapy and bile drainage, the levels of sTREM-1 decline earlier than other indexes: sTREM-1 and WBC levels decline to normality on day 8 and day 10 separately, PCT and CRP do not return to normal until the last day; there is a significant difference between the levels of sTREM-1 on day 1 and day 2 (*P* < 0.05). In other words, the concentration of sTREM-1 can be used as a monitoring indicator of treatment effectiveness. However, both Lee et al. and Prkno et al. have found that the antibiotic strategy guided by PCT shortens the duration of antibiotic treatment [29, 34]; there is a rapid response of PCT and WBC to the therapy in Magrini et al.'s research [35]; Rhee has figured out that PCT levels decline rapidly with resolution of inflammation [36]. We suspect that when operating ERCP, it will create a pressure that pushes the bile upward and causes the backflow of bacteria or endotoxins within the blood flow, leading to less obvious decline of WBC, CRP, and PCT. Some studies have also reported that transient sepsis is reported in 3~27% of patients after ERCP [13, 37]. In view of this, there are opinions that prophylactic antibiotics reduce sepsis and appear to prevent cholangitis and septicaemia in patients undergoing ERCP [38]. Since patients with AC in our hospital have not been given antibiotic therapy when they are undergoing ERCP, their levels of WBC, CRP, and PCT maintained high levels. Transient sepsis might increase gene expression of TREM-1 as well, but circulating sTREM-1 levels are not influenced. Some research has also reported the inconsistency between TREM-1 expression levels and sTEM-1 levels [39, 40].

In our study, we have observed that sTREM-1 has not been consistent with WBC when diagnosing sepsis and observing the curative effect. In sepsis, the upregulation of TREM-1 by bacterial lipopolysaccharide (LPS) magnifies the inflammatory reaction [32], leading to the improvement of sensitivity of diagnosing inflammation. In Group C (inpatients without AC or other infections), there is no correlation between WBC and sTREM-1 (*P* > 0.05). This might be caused by almost no gene expression of TREM-1 and no release of sTREM-1 in healthy individuals. In Group A and Group B, the correlation between WBC and sTREM-1 is not particularly strong, with correlation coefficient (*r*) of 0.4~0.8 (*P* < 0.01). We speculate that the degranulation and oxidative bursting by neutrophils after AC might increase in the number of free TREM-1. Thus, the measurable sTREM-1 might increase to some degree, which causes the decrease of correlation between WBC and sTREM-1. On the other hand, Dimopoulou et al. and Marioli et al. have found that serum kinetics of sTREM-1 do not follow changes of the expression of TREM-1 [19, 40]. In patients with acute cholangitis, it has been found that upon progression of the disease, the transcripts of TREM-1 gene decrease [32]. Therefore, levels of sTREM-1 are determined by more complex mechanisms. We have also found that the correlation of WBC and sTREM-1 lasts longer in Group A than Group B. It might be related to the persistent existence of inflammatory mediators in sepsis. Then, the generation of sTREM-1 keeps at a rather high level.

This study has certain limitations. First, the number of patients enrolled is limited. Second, patients of Group A have both positive blood and bile culture, and patients of Group B only have positive bile blood. Further studies need to be based upon a larger survey sample to include patients with both negative blood culture and positive bile culture. Third, we have excluded AC patients transferred to the intensive care unit (ICU) due to worsening condition, leading to the 0.0% mortality rate of this study. Further studies need to cover patients in ICU and explore the correlation between mortality and the biomarkers measured. Fourth, this is a single-center study. It is necessary for us to take a prospective study including a large number of patients from hospitals in various settings to validate the prediction of these biomarkers for the diagnosis of AC with or without sepsis. Fifth, we have not tested the gene expression of TREM-1 and inflammatory mediators related to the release of sTREM-1. So, we cannot explore the synthesis and release mechanisms of sTREM-1, as well as the kinetics of it.

## 5. Conclusion

We recommend that CRP is considered as a useful diagnostic tool for inflammation or infection, but a less accurate prognostic one for sepsis. PCT alone is well used for diagnosing AC with sepsis; meanwhile, sTREM-1 and temperature should be taken into account. sTREM-1 has great value to monitor patients' response to antimicrobial therapy and biliary drainage. Additionally, the correlation between WBC and sTREM-1 is not very strong in this study. Since our data is quite promising but not strong enough, more research is needed.

## Figures and Tables

**Table 1:** Demographic data and comorbidities of 141 subjects enrolled in this study.

Characteristics		Group A (*n* = 29)	Group B (*n* = 36)	Group C (*n* = 76)
Male/female		17/12	15/21	36/40
Mean age (y)		65 (41 → 83)	68 (37 → 82)	66 (39 → 81)
Duration of hospitalization		14 (10 → 23)	15 (12 → 21)	11 (9 → 21)
Smoking		9 (31.0%)	12 (33.3%)	25 (32.9%)
Alcohol consumption		10 (34.5%)	12 (33.3%)	25 (32.9%)
Type 2 diabetes mellitus		4 (13.8%)	6 (16.6%)	45 (59.2%)
Essential hypertension		13 (44.8%)	15 (41.7%)	31 (40.8%)
Microbiology^∗^				
Gram-negative organisms	Escherichia coli	11 (37.9%)	15 (41.6%)	—
	Klebsiella spp.	3 (10.4%)	4 (11.1%)	—
	Pseudomonas spp.	4 (13.8%)	3 (8.3%)	—
	Enterobacter spp.	2 (6.9%)	3 (8.3%)	—
	Acinetobacter spp.	1 (3.4%)	1 (2.8%)	—
	Others	1 (3.4%)	1 (2.8%)	—
Gram-positive organisms	Enterococcus spp.	5 (17.3%)	6 (16.7%)	—
	Streptococcus spp.	2 (6.9%)	1 (2.8%)	—
	Staphylococcus spp.	0 (0.0%)	1 (2.8%)	—
	Others	0 (0.0%)	1 (2.8%)	—

Group A: AC patients with sepsis; Group B: AC patients without sepsis; Group C: individuals without AC or other infections. ^∗^The microbiology results of culture in Group A were from blood and bile; the microbiology results of culture in Group B were from bile.

**Table 2:** Laboratory values of WBC, CRP, PCT, T, and sTREM-1 in three groups of each day.

	WBC (×10^9^/L)	CRP (mg/L)	PCT (ng/mL)	T (°C)	sTREM-1 (pg/mL)
Group A					
Day 1	14.3 (9.7-17.1)	133.0 (66.0-178.0)	12.78 (3.92-30.48)	37.5 (37.2-38.0)	479.0 (321.0-643.0)
Day 2	12.0 (8.9-14.5)	134.0 (99.0-187.0)	7.31 (2.54-25.90)	37.6 (37.1-38.0)	378.9 (234.5-465.0)
Day 3	9.9 (8.0-13.2)	103.0 (87.0-148.2)	2.27 (1.23-10.57)	37.2 (37.0-37.4)	264.0 (187.0-353.0)
Day 5	8.1 (6.8-10.5)	68.0 (44.0-95.3)	0.87 (0.67-2.17)	37.0 (37.0-37.2)	191.0 (143.0-287.0)
Day 8	7.3 (6.4-8.3)	25.0 (17.0-51.0)	0.35 (0.21-0.53)	37.0 (36.8-37.0)	137.5 (107.5-209.0)
Day 10	6.6 (5.7-7.3)	18.3 (11.0-31.0)	0.29 (0.21-0.39)	36.9 (36.6-37.0)	103.0 (87.0-160.2)
Group B					
Day 1	13.4 (8.9-16.2)	120.5 (88.0-164.0)	1.30 (0.40-7.60)	37.0 (36.6-37.4)	331.3 (197.6-413.5)
Day 2	10.6 (8.4-13.2)	92.5 (62.8-135.3)	0.70 (0.20-5.30)	37.4 (37.0-37.8)	229.7 (168.0-300.3)
Day 3	8.9 (6.7-10.6)	60.0 (37.8-95.0)	0.50 (0.20-1.70)	37.0 (36.9-37.2)	179.6 (141.4-245.2)
Day 5	7.3 (6.1-8.8)	26.0 (15.5-58.3)	0.20 (0.10-0.70)	37.0 (36.8-37.0)	157.7 (118.9-203.6)
Day 8	7.0 (6.0-7.9)	9.5 (5.0-21.3)	0.10 (0.10-0.30)	36.9 (36.7-37.0)	138.0 (114.0-182.2)
Day 10	7.0 (6.1-7.6)	6.5 (4.0-16.0)	0.10 (0.10-0.20)	37.0 (36.8-37.0)	140.8 (110.2-169.2)
Group C					
Day 1	6.1 (4.8-7.7)	3.3 (1.9-5.0)	0.14 (0.06-0.23)	36.9 (36.6-37.2)	125.4 (83.8-195.1)
Day 2	6.2 (4.9-7.8)	2.9 (1.8-4.4)	0.11 (0.06-0.23)	36.9 (36.7-37.1)	113.7 (75.1-192.2)
Day 3	6.4 (5.0-7.6)	2.8 (1.7-3.7)	0.11 (0.06-0.18)	36.9 (36.6-37.2)	113.0 (70.5-195.1)
Day 5	6.1 (5.1-7.7)	3.3 (1.9-5.0)	0.18 (0.09-0.29)	37.0 (36.8-37.2)	117.6 (62.2-185.1)
Day 8	6.2 (5.1-7.6)	2.9 (2.2-4.4)	0.15 (0.06-0.23)	36.9 (36.7-37.2)	120.5 (58.2-196.0)
Day 10	6.5 (5.2-7.7)	3.4 (2.1-4.5)	0.14 (0.06-0.23)	37.0 (36.7-37.1)	120.5 (67.3-196.2)

Group A: AC patients with sepsis; Group B: AC patients without sepsis; Group C: individuals without AC or other infections. Values are presented as median (interquartile range).

**Table 3:** Sensitivity and specificity of WBC, CRP, PCT, sTREM-1, and T for distinguishing Group B from Group C.

Index	AUC^ROC^	Cut-off	Sensitivity	Specificity	95% CI	PPV (%)	NPV (%)	LR(+)	LR(-)
WBC	0.859	8.95	0.750	0.974	0.759-0.958	93.1	89.2	28.85	0.26
CRP^∗^	1.000	9.45	1.000	1.000	1.000-1.000	100.0	100.0	—	0.00
PCT	0.902	0.345	0.778	0.921	0.835-0.969	82.4	89.7	9.85	0.24
T	0.574	37.25	0.417	0.868	0.446-0.702	60.0	75.9	3.16	0.67
sTREM-1	0.868	229.65	0.694	0.868	0.799-0.937	71.4	85.7	5.26	0.35

Group B: AC patients without sepsis; Group C: individuals without AC or other infections. AUC^ROC^: area under ROC curves; 95% CI: 95% confidence interval; PPV: positive predictive value; NPV: negative predictive value; LR(+): positive likelihood ratio; LR(-): negative likelihood ratio. ^∗^CRP showed the highest AUC (*z* test, *P* < 0.05) to diagnose AC without sepsis.

**Table 4:** Sensitivity and specificity of WBC, CRP, PCT, sTREM-1, and T for distinguishing Group A from Group C.

Index	AUC^ROC^	Cut-off	Sensitivity	Specificity	95% CI	PPV (%)	NPV (%)	LR(+)	LR(-)
WBC	0.939	9.25	0.828	1.000	0.881-0.997	100.0	93.8	—	0.17
CRP	1.000^∗^	12.95	1.000	1.000	1.000-1.000	100.0	100.0	—	0.00
PCT	0.975^∗∗^	0.675	0.966	1.000	0.926-1.000	100.0	98.7	—	0.03
T	0.838	37.25	0.724	0.868	0.735-0.941	67.7	89.2	5.48	0.32
sTREM-1	0.971	255.2	0.931	0.908	0.944-0.998	79.4	97.2	10.12	0.08

Group A: AC patients with sepsis; Group C: individuals without AC or other infections. AUC^ROC^: area under ROC curves; 95% CI: 95% confidence interval; PPV: positive predictive value; NPV: negative predictive value; LR(+): positive likelihood ratio; LR(-): negative likelihood ratio. ^∗^CRP and PCT showed the higher AUC^ROC^ (*z* test, *P* < 0.05). ^∗∗^The AUC^ROC^ of CRP and PCT had no significant difference (*z* test, *P* > 0.05).

**Table 5:** Sensitivity and specificity of single indexes and different index combinations for distinguishing Group A from Group B.

Index	AUC^ROC^	Cut-off	Sensitivity	Specificity	95% CI	PPV (%)	NPV (%)	LR(+)	LR(-)
WBC	0.567	9.35	0.828	0.417	0.426-0.707	53.3	75.0	1.42	0.41
CRP	0.513	148.5	0.483	0.694	0.365-0.662	56.0	62.5	1.58	0.74
PCT^∗^	0.758	3.83	0.759	0.722	0.639-0.876	68.7	78.8	2.73	0.33
T	0.750	37.35	0.655	0.722	0.631-0.869	65.5	72.2	2.36	0.48
sTREM-1^∗∗^	0.737	446	0.586	0.861	0.616-0.857	77.3	72.1	4.22	0.48
WBC & CRP^∗∗^	0.572	0.400	0.793	0.417	0.431-0.712	52.3	71.4	1.36	0.50
WBC & PCT^∗∗^	0.745	0.350	0.793	0.722	0.624-0.866	69.7	81.2	2.85	0.29
WBC & T^∗∗^	0.741	0.441	0.655	0.750	0.622-0.861	67.9	73.0	2.62	0.46
WBC & sTREM-1^∗∗^	0.784	0.610	0.552	0.944	0.669-0.898	88.9	72.3	9.86	0.47
CRP & PCT^∗∗^	0.76	0.351	0.793	0.694	0.642-0.877	67.6	80.6	2.59	0.30
CRP & T^∗∗^	0.735	0.413	0.724	0.750	0.612-0.857	70.0	77.1	2.90	0.37
CRP & sTREM-1^∗∗^	0.778	0.510	0.621	0.833	0.664-0.891	75.0	73.2	3.72	0.45
PCT & T^∗∗^	0.774	0.379	0.759	0.694	0.659-0.889	66.7	78.1	2.48	0.35
PCT & sTREM-1^∗∗^	0.745	0.487	0.586	0.861	0.625-0.865	77.3	72.1	4.22	0.48
T & sTREM-1^∗∗^	0.797	0.437	0.724	0.806	0.686-0.908	75.0	78.4	3.73	0.34
WBC & CRP & PCT^∗∗^	0.744	0.354	0.690	0.722	0.626-0.862	66.7	74.3	2.48	0.43
WBC & CRP & T^∗∗^	0.740	0.417	0.724	0.750	0.618-0.863	70.0	77.1	2.90	0.37
WBC & CRP & sTREM-1^∗∗^	0.791	0.515	0.621	0.833	0.682-0.901	75.0	73.2	3.72	0.45
WBC & PCT & T^∗∗^	0.778	0.330	0.828	0.611	0.667-0.889	63.2	81.5	2.13	0.28
WBC & PCT & sTREM-1^∗∗^	0.781	0.603	0.552	0.944	0.665-0.897	88.9	72.3	9.86	0.47
WBC & T & sTREM-1^∗∗^	0.820	0.461	0.759	0.778	0.718-0.921	73.3	80.0	3.42	0.31
CRP & PCT & T^∗∗^	0.780	0.419	0.690	0.778	0.663-0.896	71.4	75.7	3.11	0.40
CRP & PCT & sTREM-1^∗∗^	0.775	0.383	0.793	0.694	0.660-0.890	67.6	80.6	2.59	0.30
CRP & T & sTREM-1^∗∗^	0.803	0.464	0.690	0.833	0.692-0.913	76.9	76.9	4.13	0.37
PCT & T & sTREM-1^∗∗^	0.793	0.382	0.793	0.694	0.682-0.904	67.6	80.6	2.59	0.30
WBC & CRP & PCT & T^∗∗^	0.786	0.455	0.655	0.861	0.675-0.897	79.2	75.6	4.71	0.40
WBC & CRP & PCT & sTREM-1^∗∗^	0.797	0.469	0.655	0.833	0.688-0.906	76.0	75.0	3.92	0.41
WBC & CRP & T & sTREM-1^∗∗^	0.833	0.653	0.552	0.944	0.736-0.930	88.9	72.3	9.86	0.47
WBC & PCT & T & sTREM-1^∗∗^	0.825	0.467	0.690	0.861	0.724-0.926	80.0	77.5	4.96	0.36
CRP & PCT & T & sTREM-1^∗∗^	0.797	0.468	0.690	0.806	0.685-0.909	74.1	76.3	3.56	0.38
WBC & CRP & PCT & T & sTREM-1^∗∗^	0.824	0.615	0.586	0.944	0.723-0.925	89.5	73.9	10.46	0.44

Group A: AC patients with sepsis; Group B: AC patients without sepsis. AUC^ROC^: area under ROC curves; 95% CI: 95% confidence interval; PPV: positive predictive value; NPV: negative predictive value; LR(+): positive likelihood ratio; LR(-): negative likelihood ratio. ^∗^PCT showed the highest AUC^ROC^ (*z* test, *P* < 0.05) to diagnose sepsis in patients with AC, followed by T and sTREM-1. ^∗∗^Different combination of indexes did not enhance the diagnostic power (*z* test, *P* > 0.05).

**Table 6:** Correlation coefficients (*r*) between WBC and sTREM-1 in three groups.

Group	Day 1	Day 2	Day 3	Day 5	Day 8	Day 10
Group A^a^	0.623^∗^	0.744^∗^	0.663^∗^	0.493^∗^	0.498^∗^	0.178
Group B^b^	0.678^∗^	0.607^∗^	0.432^∗^	0.231	0.202	0.155
Group C	0.153	0.009	0.123	0.068	0.110	0.014

^∗^ *P* < 0.01; Group A: AC patients with sepsis; Group B: AC patients without sepsis; Group C: individuals without AC or other infections. ^a^The correlation coefficients in Group A from day 1 to day 8 had no significant difference with each other (*P* > 0.05). ^b^The correlation coefficients in Group B from day 1 to day 3 had no significant difference with each other (*P* > 0.05).

## Data Availability

The biomarkers' data used to support the findings of this study are available from the corresponding author upon request.

## Abbreviations

AC: Acute cholangitis
BTI: Biliary tract infection
WBC: White blood cell
CRP: C-reactive protein
PCT: Procalcitonin
sTREM-1: Soluble triggering receptor expressed on myeloid cells 1
T: Temperature
ERCP: Endoscopic retrograde cholangiopancreatography
ROC: Receiver operating characteristic
AUC: Area under curves
CI: Confidence intervals
PPV: Positive predictive value
NPV: Negative predictive value
LR(+): Positive likelihood ratio
LR(-): Negative likelihood ratio.
